# Letter to the editor: ‘Comparative dose-response study on the infusion of norepinephrine combined with intravenous ondansetron versus placebo for preventing hypotension during spinal anesthesia for cesarean section – a randomised controlled trial’

**DOI:** 10.1097/JS9.0000000000001255

**Published:** 2024-03-04

**Authors:** Hanliang Fan, Ting Zheng, Peng Ye, Xiaochun Zheng

**Affiliations:** a Department of Anesthesiology, Shengli Clinical Medical College of Fujian Medical University, Fujian Provincial Hospital; b Fujian Emergency Medical Center, Fujian Provincial Key Laboratory of Emergency Medicine, Fujian Provincial Key Laboratory of Critical Care Medicine, Fujian Provincial Co-Constructed Laboratory of “Belt and Road”, Fuzhou, People’s Republic of China

*Dear Editor*,

In a recent study conducted by Sheng ZM *et al*.^1^, it was demonstrated that intravenous administration of ondansetron at 0.1 mg/kg prior to spinal anesthesia significantly reduced the necessary dosage of prophylactic norepinephrine infusion in patients undergoing elective cesarean delivery. The researchers showed that the effective doses for hypotension prevention (ED50 and ED90) of norepinephrine infusion during spinal anesthesia were decreased by ~36 and 35%, respectively, when ondansetron was administered 10 min beforehand. These findings offer valuable recommendations for determining the initial norepinephrine infusion dose, with or without adjunctive prophylactic ondansetron, to mitigate hypotension associated with spinal anesthesia in cesarean delivery. However, there remain several unresolved questions in the article that would benefit from further elucidation by the authors.

Firstly, in this study, patients were randomly assigned to one of five prophylactic norepinephrine infusion groups: 0.02, 0.04, 0.06, 0.08, or 0.10 µg/kg/min, with 15 participants in each subgroup. Using probit regression, ED50 and ED90 values for norepinephrine infusion were ascertained and then compared across groups to determine the effective dose for preventing hypotension during spinal anesthesia for cesarean delivery. Xiao F *et al*.^2^ ’s research similarly evaluated the effective dose of phenylephrine infusion (ED50) necessary to prevent hypotension in 50% of patients through up-down sequential analysis. It is well-recognized that the modified Dixon’s up-and-down method is more sample-efficient and a classic approach in clinical pharmacodynamics research^3,4^. The incorporation of Dixon’s method in this study raises the question whether it could lead to a reduction in the number of groups and the overall sample size. The incorporation of Dixon’s method in this study raises the question whether it could lead to a reduction in the number of groups and the overall sample size.

Secondly, the combined spinal-epidural anesthesia was administered at the L2-L3 or L3-L4 interspace, where 16 mg of hyperbaric ropivacaine was infused at a rate of 0.2 ml/s, following the anesthesiologist’s verification of a clear, free-flowing cerebrospinal fluid utilizing a needle-through-needle technique, with the patients in a left lateral position. The uniform dosage of ropivacaine across all patients may result in divergent hypotension rates due to individual variations in spinal anesthesia depth, potentially influencing the study’s outcomes^5^. Moreover, interspace selection variability for the puncture could cause different hypotension rates.

Thirdly, various studies have administered 8 mg of ondansetron, while others have used 4 mg^6^. Why not just use 4 mg ondansetron and metaraminol or phenylephrine in a randomised trial, which is large enough to deal with the known variable of obstetrics.

In conclusion, this rigorously executed clinical study furnishes valuable recommendations for determining the initial dose of norepinephrine infusion, with or without the inclusion of prophylactic intravenous ondansetron, aimed at preventing hypotension during spinal anesthesia in cesarean section.

## Ethical approval

This article does not require any human or animal subjects to acquire such approval.

## Consent

This article does not require any human or animal subjects to acquire such approval.

## Sources of funding

The authors received no financial support.

## Author contribution

X.Z.: conceptualized and review and editing; H.F., T.Z., and P.Y.: writing, made the draft, and updated it. All authors have critically reviewed and approved the final draft and are responsible for the content and similarity index of the manuscript.

## Conflicts of interest disclosure

The authors declare no conflicts of interest.

## Research registration unique identifying number (UIN)

None.

## Guarantor

All authors.

## Data availability statement

This submission does not contain any data. The manuscript is ‘letter to the editor’ and is based on previously reported data and analyses that have been cited appropriately within the text. Consequently, there are no new data associated with this article.

## Data availability statement

The datasets utilized and/or analyzed in this study can be obtained from the corresponding author upon a reasonable request.

## Provenance and peer review

Not commissioned, externally peer-reviewed.
